# Interaction between Sugarcane and Environmental Stressors: From Identification to Molecular Mechanism

**DOI:** 10.3390/plants13141973

**Published:** 2024-07-19

**Authors:** San-Ji Gao, Talha Javed

**Affiliations:** 1 National Engineering Research Center for Sugarcane, Fujian Agriculture and Forestry University, Fuzhou 350002, China; 2 Institute of Tropical Bioscience and Biotechnology, Chinese Academy of Tropical Agricultural Sciences, Haikou 571101, China; talhajaved@itbb.org.cn

An increase in the vulnerability of crops to a wide range of biotic and abiotic stresses can have a marked influence on the productivity and quality of major crops, especially sugarcane (*Saccharum* spp.). To cope with various stressful conditions, plants have evolved complex rapid response mechanisms [1,2]. The broader topic of this special issue emphasizes on the interaction between sugarcane and environmental stressors at genotypic, physio-chemical, and molecular levels. This special issue includes a total of 13 papers with 12 research papers and one review article. Papers were submitted from six countries, including Pakistan, Saudi Arabia, Egypt, Poland, Thailand, and China. These papers were grouped into four main categories: (1) comparative analysis of agronomic characters (growth, yield, and quality) among sugarcane genotypes amidst different environmental conditions; (2) microbial community analysis on sugarcane-growing soil and population genetic analysis on pathogens infecting sugarcane; (3) transcriptome analysis of sugarcane defense responses to various pathogens; and (4) transgenesis and functional analysis of sugarcane genes under different stressors. This editorial encompasses perspectives for the future vision of sugarcane improvement under climate change scenarios.

The study by Manzoor et al., investigated the influence of different ecological zones and irrigation conditions on sugarcane growth, yield, and quality in Pakistan. In general, the utilization of combined canal and tube well water as well as the combined application of potassium and boron improved sugarcane yield and quality [3]. Sajid et al. found that sugarcane genotypes S2006-US-658, HSF-240, and S2007-AUS-384 were better performers concerning growth and physiological attributes under sandy loam soil and water deficit conditions [4]. Similarly, Zhao et al., revealed that variance in sugarcane yields and sugar contents was caused by climate differences in the low-latitude plateau [5]. The observation by Wu et al. (2024) demonstrated that combined application of biochar and microplastics have potential to alter the soil nutrients and microbial community structure and function and alleviate the negative effects of micro-plastic accumulation on sugarcane biomass [6]. In general, the elite genotype breeding and soil improvement are important strategies for sugarcane adaptation under climate change scenarios.

Xu et al. analyzed viral population structure and evolutionary forces, demonstrating the phylogeny and genetic divergence among sorghum mosaic virus (SrMV) isolates infecting sugarcane. This work showed that the geographical isolation and host types are important factors promoting the genetic differentiation of SrMV populations [7]. Another study by Zhou et al., showed that significantly lower viral proliferation was observed in two cultivars ROC22 and Xuezhe under 1 mM exogenous salicylic acid (SA) application [8]. Additionally, Shan et al. revealed two non-synonymous mutation sites for amino acids located in the cap pocket of eIF4E, which might be related to the resistance to sugarcane streak mosaic virus (SCSMV) [9].

Transcriptome analysis based on the RNA-seq platform by Illumina NGS technology is commonly used for investigating sugarcane defense responses to pathogen infections. Zhou et al., revealed that most of the differentially expressed genes (DEGs) were mainly concentrated in plant-pathogen interaction and phenylpropanoid biosynthesis, which involved in SA-induced disease resistance of sugarcane in response to SrMV infection [8]. The RNA-seq dataset and RT-qPCR assay showed that the transcript levels of genes related to SA biosynthesis and signal transduction pathways were significantly upregulated in sugarcane in response to SA treatment under SrMV infection [8]. A transcriptomic study by Lohmaneeratana et al., examines the increased expression of DEGs associated with the phenylpropanoid, plant hormones, calcium, and mitogen activated protein kinase (MAPK) cascades that positively promoted defense mechanisms against *Candidatus sacchari* (a causal agent of sugarcane white leaf disease) colonization by triggering calcium and accumulation in response to plant immunity [10]. Furthermore, Zhang et al., found that the *pglA* gene from *Leifsonia xyli* subsp. *xyli* (causing ratoon stunting disease) interacted with the host protein SoSnRK1β1 (an important protein in the ABA pathway), suggesting ABA pathway is likely triggered host resistance to this pathogen [11]. Khan and colleagues also conducted the comparative transcriptomic analysis to examine the functions of sucrose regulatory genes in high- and low-sucrose sister clones of sugarcane. According to this study, the enhanced expression of sucrose phosphate synthase (SPS) in the high-sucrose clone compared to the low sucrose-clone implies that SPS is mostly responsible for the greater accumulation of sucrose [12].

Recently, molecular breeding using transgenic and/or gene-edition technologies have contributed significantly to sugarcane improvement, but a simple, robust, and efficient transgenics is a crucial step in molecular breeding [2]. Thus, Wang et al., established an efficient sugarcane transformation system via herbicide-resistant CP4-EPSPS gene selection, as evidenced by high yield of transgenic lines from calli, an improved selection method, a higher transformation efficiency, and enhanced resistance against the Roundup herbicide in transgenic sugarcane plants [13]. Additionally, the illustrated mechanisms of sugarcane defense against environmental stressors provide important clues for sugarcane genetic improvement. Wei et al., showed that reactive oxygen species (ROS) production–scavenging system is involved in sugarcane response to *Xanthomonas albilineans* (*Xa*, causing leaf scald) infection under drought stress. This study also showed that, whereas drought stress had no discernible impact on resistant cultivars, it greatly enhanced the incidence of leaf scald and *Xa* populations in susceptible cultivars. Under coupled *Xa* infection and drought stress, sugarcane (particularly susceptible cultivars) showed a weakened defense response via the ROS-producing and scavenging system as compared to *Xa* infection alone [14].

In summary, this special issue illustrated current research findings about interaction between sugarcane and environmental stressors. Future research in this area will be driven by the integration of multi-omics tools (phenomics, transcriptomes, proteomics, and epigenetics) and utilization of modern biotechnological tools for understanding the trade-off between growth and defense in sugarcane, which will provide clues for the development of breeding strategies to generate elite cultivars for meeting rising global sugar and biofuel demand.

## References

[1] Javed T., Shabbir R., Ali A., Afzal I., Zaheer U., Gao S.-J. (2020). Transcription Factors in Plant Stress Responses: Challenges and Potential for Sugarcane Improvement. Plants.

[2] Kumar T., Wang J.-G., Xu C.-H., Lu X., Mao J., Lin X.-Q., Kong C.-Y., Li C.-J., Li X.-J., Tian C.-Y. (2024). Genetic Engineering for Enhancing Sugarcane Tolerance to Biotic and Abiotic Stresses. Plants.

[3] Manzoor M., Khan M.Z., Ahmad S., Alqahtani M.D., Shabaan M., Sarwar S., Hameed M.A., Zulfiqar U., Hussain S., Ali M.F. (2023). Optimizing Sugarcane Growth, Yield, and Quality in Different Ecological Zones and Irrigation Sources Amidst Environmental Stressors. Plants.

[4] Sajid M., Amjid M., Munir H., Ahmad M., Zulfiqar U., Ali M.F., Abul Farah M., Ahmed M.A.A., Artyszak A. (2023). Comparative Analysis of Growth and Physiological Responses of Sugarcane Elite Genotypes to Water Stress and Sandy Loam Soils. Plants.

[5] Zhao Y., Yu L.-X., Ai J., Zhang Z.-F., Deng J., Zhang Y.-B. (2023). Climate Variations in the Low-Latitude Plateau Contribute to Different Sugarcane (*Saccharum* spp.) Yields and Sugar Contents in China. Plants.

[6] Wu Q., Zhou W., Chen D., Tian J., Ao J. (2024). Biochar Mitigates the Negative Effects of Microplastics on Sugarcane Growth by Altering Soil Nutrients and Microbial Community Structure and Function. Plants.

[7] Xu H.-M., He E.-Q., Yang Z.-L., Bi Z.-W., Bao W.-Q., Sun S.-R., Lu J.-J., Gao S.-J. (2023). Phylogeny and Genetic Divergence among Sorghum Mosaic Virus Isolates Infecting Sugarcane. Plants.

[8] Zhou G., Shabbir R., Sun Z., Chang Y., Liu X., Chen P. (2024). Transcriptomic Analysis Reveals Candidate Genes in Response to Sorghum Mosaic Virus and Salicylic Acid in Sugarcane. Plants.

[9] Shan H., Chen D., Zhang R., Wang X., Li J., Wang C., Li Y., Huang Y. (2023). Relationship between Sugarcane *eIF4E* Gene and Resistance against Sugarcane Streak Mosaic Virus. Plants.

[10] Lohmaneeratana K., Leetanasaksakul K., Thamchaipenet A. (2024). Transcriptomic Profiling of Sugarcane White Leaf (SCWL) Canes during Maturation Phase. Plants.

[11] Zhang X.-Q., Liang Y.-J., Zhang B.-Q., Yan M.-X., Wang Z.-P., Huang D.-M., Huang Y.-X., Lei J.-C., Song X.-P., Huang D.-L. (2024). Screening of Sugarcane Proteins Associated with Defense against *Leifsonia xyli* subsp. *xyli*, Agent of Ratoon Stunting Disease. Plants.

[12] Khan Q., Qin Y., Guo D.-J., Huang Y.-Y., Yang L.-T., Liang Q., Song X.-P., Xing Y.-X., Li Y.-R. (2024). Comparative Analysis of Sucrose-Regulatory Genes in High- and Low-Sucrose Sister Clones of Sugarcane. Plants.

[13] Wang W., Javed T., Shen L., Sun T., Yang B., Zhang S. (2024). Establishment of an Efficient Sugarcane Transformation System via Herbicide-Resistant CP4-EPSPS Gene Selection. Plants.

[14] Wei Y.-S., Zhao J.-Y., Javed T., Ali A., Huang M.-T., Fu H.-Y., Zhang H.-L., Gao S.-J. (2024). Insights into Reactive Oxygen Species Production-Scavenging System Involved in Sugarcane Response to *Xanthomonas albilineans* Infection under Drought Stress. Plants.

